# Tensile Properties of Three Selected Collagen Membranes

**DOI:** 10.1155/2019/5163603

**Published:** 2019-12-05

**Authors:** Perry Raz, Tamar Brosh, Guy Ronen, Haim Tal

**Affiliations:** ^1^Department of Periodontology and Oral Implants, The Goldschleger School of Dental Medicine, Sackler Faculty of Medicine, Tel Aviv University, Tel Aviv, Israel; ^2^Department of Oral Biology, The Goldschleger School of Dental Medicine, Sackler Faculty of Medicine, Tel Aviv University, Tel Aviv, Israel; ^3^The Goldschleger School of Dental Medicine, Sackler Faculty of Medicine, Tel Aviv University, Tel Aviv, Israel

## Abstract

**Background:**

Biological barriers are commonly used to treat alveolar bone defects and guide tissue regeneration. Understanding the biological and mechanical properties of the available membranes is crucial for selecting the one that is optimal for enhancing clinical outcomes.

**Purpose:**

To evaluate the mechanical behavior of three different collagen membranes to increasing tensile force in dry and wet conditions.

**Materials and Methods:**

Three commercially collagen membranes were selected for analysis: Bio-Gide® (Geistlich Biomaterials, Baden-Baden, Germany), Remaix™ (RX; Matricel GmbH, Herzogenrath, Germany), and Ossix Plus® (Datum Dental Biotech, Lod, Israel). Increasing tensile forces were applied on 10 dry and wet membranes of standard size via a loading machine. Force and extension values were acquired up to maximum load before failure, and maximum stress, maximum extension, and amount of energy needed for membrane tearing were analyzed. Membranes' densities were also calculated.

**Results:**

The Remaix membrane exhibited the highest values of maximum load tensile strength, maximum extension, and maximum energy required for membrane tearing, followed by Bio-Gide. Ossix Plus had the lowest scores in all these parameters. Dry membranes had the highest scores for all parameters except extension. Membrane density was directly and significantly correlated with all tested parameters.

**Conclusions:**

The study was undertaken to provide clinicians with data upon which to base the selection of collagen membranes in order to achieve optimal clinical results. It emerged that the mechanical properties of dry and wet collagen membranes were significantly different from one another. Among the 3 tested membranes, Remaix exhibited higher performance results in all the mechanical tests. Collagen membrane density seems to have a significant influence upon mechanical resistance. These findings may also guide manufacturers in improving the quality of their product.

## 1. Introduction

Biological barriers are commonly used to promote guided tissue regeneration (GTR). Regenerative periodontal therapy often aims to restore the periodontal attachment apparatus that had been destroyed due to periodontal disease [1–3]. Treatment of alveolar bone defects (referred to as guided bone regeneration (GBR)) is similarly supported by such barrier membranes [4]. Understanding the biological and mechanical properties of the available membranes is crucial for selecting the one that is optimal for enhancing clinical outcomes. Regardless of whether the procedure is GTR or GBR, the barrier membrane should exhibit all or most of the following qualities:

- Biocompatibility that will allow integration of the barrier with the host tissues without eliciting inflammatory responses
- A suitable degradation profile to match that of new tissue formation
- Appropriate mechanical and physical properties to allow its placement in vivo
- Sufficient sustained strength to avoid membrane collapse and function as a barrier
- Cell occlusiveness for the ability to act as a cell barrier, i.e., to exclude undesirable cells from entering the secluded space [5]

Human studies have provided evidence of the effectiveness of regenerative treatment of intrabony defects with collagen membranes, with or without the addition of bone substitutes [6–13]. Combining collagen membrane with bone substitutes may act as a supporting scaffold that prevents the barrier from collapsing, especially in noncontained intrabony defects [10]. Data from systematic reviews have suggested that GTR indeed improves clinical outcomes [11–13]. For the reconstruction of an alveolar bone ridge for implant placement using GBR techniques, the membrane is placed over the defect site, isolating it from the surrounding soft tissues and encouraging population of the site with primarily bone progenitor cells, osteoblasts, and other regenerative factors [14].

Membranes may be classified according to the chemistry of the manufactured material, such as synthetic polymers (e-polytetrafluoroethylene (PTFE)), polylactic acid (PLA), polyglycolic acid, synthetic or native collagen, metals (titanium), and inorganic compounds (calcium sulfate). They may also be classified into resorbable and nonresorbable membranes [15]. Collagen membranes are the most popular barriers used in both GTR and GBR procedures, and the collagen for such purposes is available from different animal sources, especially porcine and bovine [13]. The main advantages of resorbable collagen membranes are that collagen is a major constituent of the natural extracellular matrix (ECM) and an important alternative to synthetic polymers due to its excellent cell affinity and biocompatibility [16]. Importantly, the use of nonresorbable membranes increases the risk of membrane exposure to bacterial colonization, thereby possibly inhibiting healing [17, 18].

Various cross-linking techniques have been developed to control the absorption time of collagen membranes, including ultraviolet light, hexamethylene di‐isocyanate, glutaraldehyde plus irradiation, and diphenylphosphoryl azide [19]. The cross-linking of collagen with aldehyde sugars, especially ribose nonenzymatic glycation, produces collagen barriers that are more resistant to resorption, thus allowing a slower degradation rate [20]. Immobilization of membranes is essential for establishing the proper environment necessary for clot stabilization, cell proliferation and differentiation, and tissue regeneration. Stabilizing of membranes is achieved by sutures and/or miniscrews and pins [21].

It has been claimed that the use of barrier membranes with insufficient stability and inadequate space maintenance in GBR may lead to the displacement of the grafts by the stress generated by the peripheral soft tissues, resulting in the reduction of new bone growth [22]. Therefore, the ideal membrane should be sufficiently rigid to withstand the compression of overlying soft tissues and possess the required degree of plasticity for being easily contoured and molded into the desired shape to conform to the defect. A balance between these mechanical properties is an advantage.

Due to the sparse data available on the mechanical properties of collagen membranes, the aim of this study was to compare the resistance of three commercially available ones to gradually increasing tensile force in dry and wet conditions. The study was undertaken to provide clinicians and manufacturers with data which might improve the clinical selection of a membrane and guide the manufacturing process for yielding better results by providing improved products.

## 2. Materials and Methods

Three commercially available collagen membranes which are used for GBR and/or GTR procedures were selected for the study: two different non-cross-linked collagen membranes, Bio-Gide® (Geistlich Biomaterials, Baden-Baden, Germany) and Remaix™ (RX; Matricel GmbH, Herzogenrath, Germany), and one cross-linked collagen membrane, Ossix Plus® (Datum Dental Biotech, Lod, Israel).

Bio-Gide is a bilayer barrier made of porcine dermis type I and III collagen with one compact layer facing the soft tissue and acting as a tissue barrier and a second layer that is spongy and porous to allow bone tissue integration [23]. Remaix is composed of a network of highly purified non-cross-linked porcine collagen fibers intermingled with porcine elastin fibers. Ossix Plus is a synthetic ribose-induced cross-linked membrane made of porcine tendon type I collagen [24].

The membranes, from Tel Aviv School of Dental Medicine, were cut to pieces of standard size (5 × 15 mm) by means of a custom-made guillotine. The mean thickness and volume of each membrane were calculated by measuring the thickness of each membrane at 3 different points by means of an analogue caliper (Kafer F1101/30) with a resolution of 1 *μ*m and an error range of 3 *μ*m [25]. Specimens were weighed by an analytical balance (Sartorius TE64 Talent Analytical Balance), and their density was calculated by dividing each specimen's weight by its volume. To achieve wet samples, the membranes were soaked in saline solution according to the manufacturer's instructions as follows: Ossix Plus for 3 min, Bio-Gide for 30 min, and Remaix for a few seconds until it was damp. Ten membranes of each group were clamped to a loading machine (Instron, Model 4502, Buckinghamshire, England) equipped with a 100 N load cell, where the distance between the clamps was maintained at 10 mm. Equal samples of each group (*n* = 10) were tested in dry and wet conditions at a crosshead speed of 1 mm/min until the point of membrane tearing. Tensile force (TF) was continuously recorded, and the TF vs. membrane extension result was acquired automatically. Four parameters were considered for mechanical analysis:

1. Maximal load (N) was measured and recorded at the extreme loading point, just before tearing
2. Tensile strength (maximum stress, MPa) was calculated as the maximal load divided by the cross-sectional area of each specimen
3. Maximal extension (mm) was measured at the point of maximal load
4. The energy applied during loading (J) was calculated as the area under the load extension curve up to maximal load

### 2.1. Statistical Analysis

The square roots of the parameters were considered for the statistical analysis in order to obtain normal distribution of the results. Comparisons between the parameters were analyzed by analysis of variance with repeated measures with 2 factors: type (manufacturer) and condition (dry or wet) followed by a post hoc test. The *t*-test was applied to verify the differences between tearing forces for the 3 groups. Correlations between the different parameters and the density were tested using the Pearson correlation. Differences were accepted as being significant at a *p* value < 0.05 using SPSS ver.20.

## 3. Results

**Figure 1:**
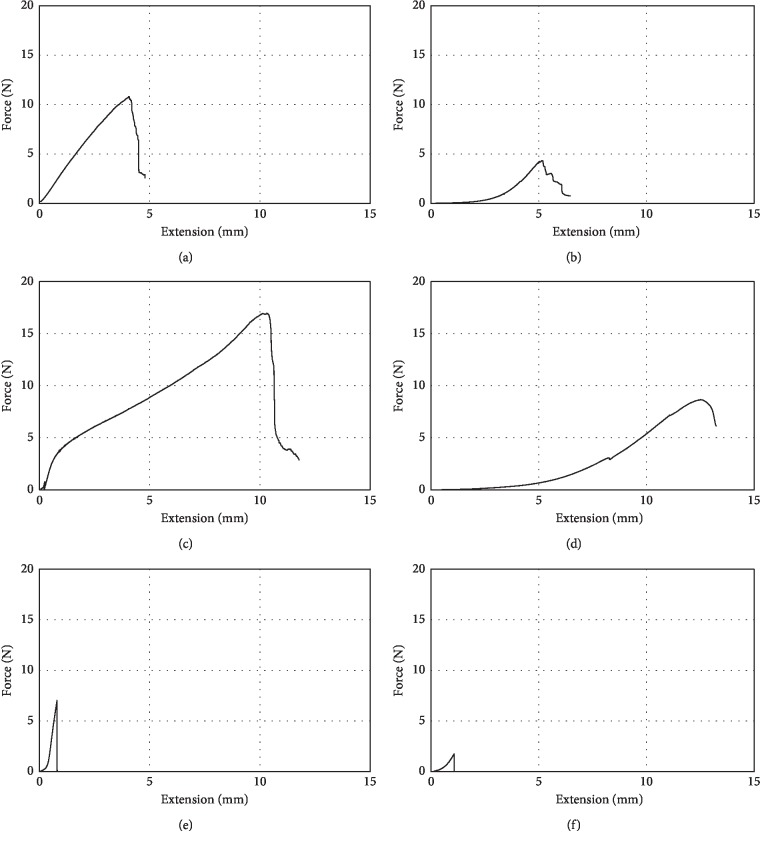
Characteristic force-extension diagrams: (a) Bio-Gide dry, (b) Bio-Gide wet, (c) Remaix dry, (d) Remaix wet, (e) Ossix Plus dry, and (f) Ossix Plus wet.

The non-cross-linked membranes, Remaix and Bio-Gide, exhibited nonlinear behavior during loading in dry and wet conditions, whereas the cross-linked membrane, Ossix Plus, exhibited linear behavior only in the dry condition. Therefore, the energy applied during loading was considered rather than the slope of the loading curve of the membranes (representing elastic modulus) (Figure 1).

Both the type of the membrane and their condition (dry or wet) significantly affected the measured parameters in the tensile test (*p* < 0.001, two-way ANOVA).

**Figure 2:**
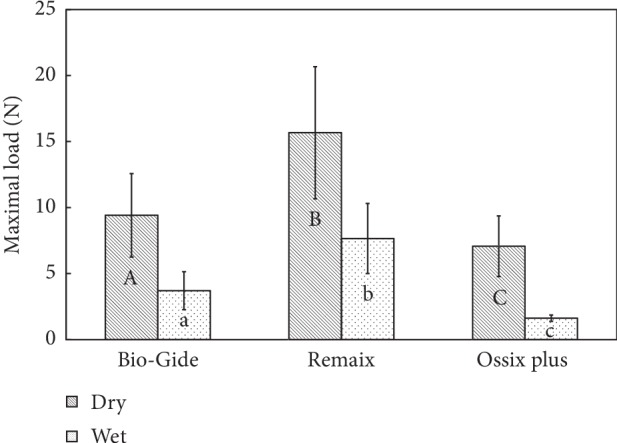
Comparison between the maximum loads that were needed for reaching the tearing/failure point of the membranes in dry and wet conditions (*N* = 10). Groups with different letters, upper case and lower case, per material and between materials are significantly different (*p* < 0.05).

Figure 2 presents the mean maximal load of all 3 membrane groups. The maximal load was significantly higher for membranes in the dry condition compared to the wet condition (*p* < 0.001): Remaix (dry: 15.67 ± 5 N, wet: 7.66 ± 2.65 N) and Bio-Gide (dry: 9.41 ± 3.16 N, wet: 3.7 ± 1.43 N), with Ossix Plus presenting the lowest maximal force (dry: 7.07 ± 2.29 N, wet: 1.63 ± 0.24 N).

**Figure 3:**
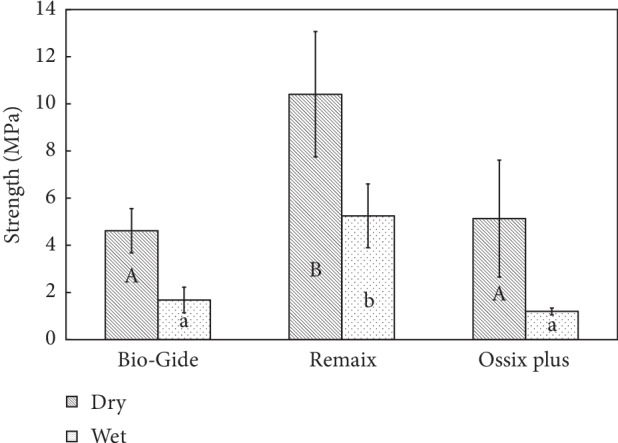
Comparison of maximum stress (maximum load to the cross section area, MPa) measured between the membranes and in dry and wet conditions (*N* = 10). Groups with different letters, upper case and lower case, per material and between materials are significantly different (*p* < 0.05).

Figure 3 presents the mean tensile strength of all 3 membrane groups. Remaix scored the highest tensile strength (dry: 10.4 ± 2.66 MPa, wet: 5.25 ± 1.35 MPa), followed by Bio-Gide (dry: 4.6 ± 0.94 MPa, wet: 1.68 ± 0.54 MPa) and Ossix Plus (dry: 5.13 ± 2.48 MPa, wet: 1.2 ± 0.14 MPa); however, Ossix Plus did not show a significant difference compared with Bio-Gide (*p* > 0.05).

**Figure 4:**
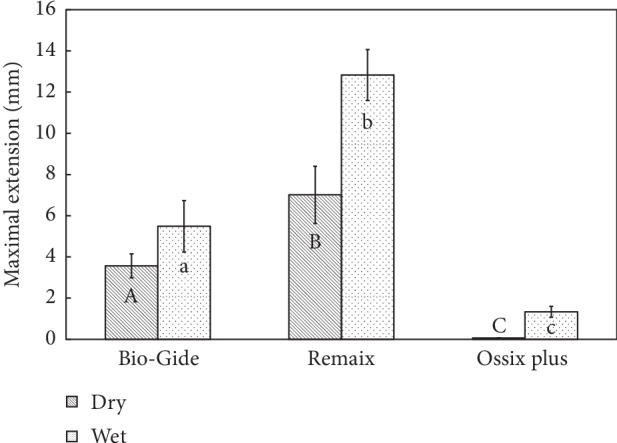
Comparison between the maximum extensions of the membranes at the tearing/failure point and between dry and wet conditions (*N* = 10). Groups with different letters, upper case and lower case, per material and between materials are significantly different (*p* < 0.05).

The extension of the membranes at maximal load is presented in Figure 4. All the wet membranes extended to a significantly longer distance compared to the dry ones (Tukey's test *p* < 0.05). Remaix produced the longest extension under both dry and wet conditions (dry: 7.01 ± 1.38 mm, wet: 12.83 ± 1.23 mm) followed by Bio-Gide (dry: 1.9 ± 0.15 mm, wet: 2.3 ± 0.25 mm), while Ossix Plus exhibited extension values of 0.06 ± 0.00 mm and 1.33 ± 0.26 mm in dry and wet conditions, respectively. The membrane type was significantly associated with the maximum extension in both the dry and wet conditions (*p* < 0.01).

**Figure 5:**
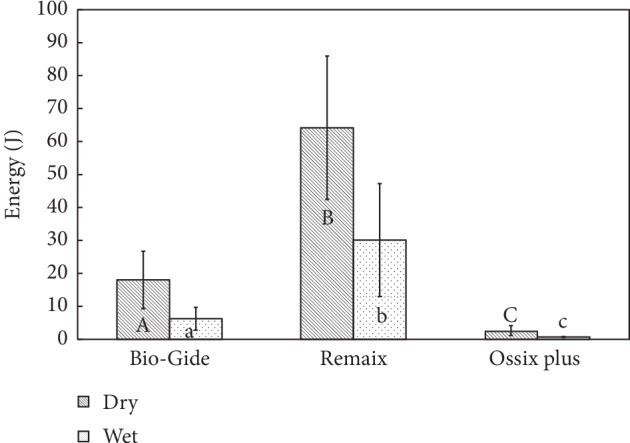
Comparison between the energy required for membrane tearing/failure which was calculated as the area under the curve (AUC). The energy (J) required for tearing membranes in dry and wet conditions (*N* = 10). Groups with different letters, upper case and lower case, per material and between materials are significantly different (*p* < 0.05).

Figure 5 depicts the energy required for membrane tensile test. The dry membranes required significantly more energy to tear than the wet ones (*p* < 0.001). The energy needed for tearing the Remaix membrane was the highest (dry: 64.15 ± 21.77 J, wet: 30.11 ± 17.01 J) followed by Bio-Gide (dry: 4.12 ± 1.05 J, wet: 2.41 ± 0.68 J) and Ossix Plus (dry: 2.65 ± 1.51 J, wet: 0.712 ± 0.11 J).

The average physical data of the tested membranes (i.e., thickness and volume) are presented in Table 1. Membrane density was also calculated, and it was found to be similar for both the wet and dry membranes. Membrane thickness was found to be statistically significant between Bio-Gide and Remaix (Bio-Gide was 47% thicker) and between Bio-Gide and Ossix Plus (Bio-Gide was 58% thicker). In dry conditions, Remaix was denser than both Bio-Gide and Ossix Plus (0.43 ± 0.028 mg/mm^3^, 0.33 ± 0.04 mg/mm^3^, and 0.22 ± 0.03 mg/mm^3^), respectively (Table 1).

Correlations between the physical parameters and the mechanical data were performed on all the examined membranes, wet or dry (*n* = 30), in order to determine the most important physical character for improving the mechanical performance of membrane. There were significantly high correlations (*p* < 0.01) between the density of the membrane and all measured mechanical parameters in all 3 groups (Table 2).

## 4. Discussion

The primary aim of this study was to determine and analyze the tensile strength of 3 commercially available collagen membranes. Two of them, Remaix and Bio-Gide, are made from native not-cross-linked collagen, while Remaix contains elastin fibers, and the third, Ossix Plus, is made from sugar-induced cross-linked collagen. The results of this analysis revealed clear-cut differences between the examined membranes. The parameters that were measured included maximum load, maximum stress, maximal extension, and energy required for membrane tearing, and Remaix exhibited the highest values for all of them, followed by Bio-Gide and then Ossix Plus. These characteristics applied to both dry and wet membranes. The tensile strengths for all the dry membranes were higher than those of the wet ones, with the exception of the extension parameter. Membrane density correlated positively and significantly with all the other tested parameters. The surface area also correlated positively but not to a level of significance. Membranes' thickness did not have a significance influence on the differences between the examined mechanical properties as indicated by the finding that the Remaix membrane was not the thickest membrane but rather had superior mechanical properties.

While there are no published comparative data on these 3 specific membranes, the current findings are in accordance with Bozkurt et al. [24] who examined the mechanical properties of Bio-Gide and Remaix. Those authors claimed that the tensile stress of the Remaix membrane in dry conditions (“stress at break”) was twice that of Bio-Gide. They suggested that the improved mechanical properties of the Remaix membrane may extend the current range of indications for collagen membranes and stressed that high tensile strength and stability of membranes are required for combined horizontal and vertical bone ridge augmentation, where relatively large defects must be bridged and the membrane must be stabilized by being fixed with pins to the surrounding bone. A similar tear load of 1.84 N was found for Bio-Gide membrane by Ortolani et al. who had applied a mechanical test [25].

The current results are also in accordance with Roeder et al. [26] who investigated the mechanical properties of the collagen type I ECM stress-strain curves of assembled matrices of similar shapes in vitro. They observed that collagen matrices exhibited nonlinear stress-strain curves with 3 distinct regions. The first was a region of small strains called the “toe”' region, which corresponds to the removal of a crimp in the collagen fibrils first at the fibrillar level and then at the molecular level. The second was a “linear” region, representing the stiffness of the collagen fibrils which increases considerably with extension. This region has been associated with stretching of the collagen triple helices or of the cross-links between the helices, implying a side-by-side gliding of neighboring molecules. The third was the ‘‘failure” region, representing disruption of the fibril structure. Additionally, the stress-strain response of the collagen matrices was shown to be sensitive to strain rate, a characteristic of viscoelastic materials. Finally, the mechanical nature of collagen matrices was shown to be consistent with the properties of their fibrils, which is their main structural component.

The differences between the 3 collagen membranes that emerged from the results of this study may be explained by the different structure of a given membrane's material. The “Glymatrix” technology used for producing the ribose-induced cross-linking of the Ossix Plus membrane was probably responsible for the increased fragility and brittleness of the synthetic membrane, whereas the native, non-crossed-linked collagen of Bio-Gide and Remaix had more elasticity, a feature that would improve the ability of handling as well as the adaptation to more irregular surfaces. In addition, Bio-Gide has individual collagen fibrils interlaced to form coarse collagen strands [27].

According to the manufacturer's instructions for use, the membranes should be placed in saline before placement in situ. However, Coïc et al. [28], found that extensive moistening considerably alters the mechanical properties of the membranes, an observation that is in accordance with the present results. In contrast, this does not happen to the Ossix Plus crossed-linked membrane, which has a fragile response to the application of tensile forces in either the dry or wet condition.

Depalle et al. [29] claimed that the mechanical response of cross-linked fibrils exhibits a 3-phase pattern of behavior: (i) an initial elastic deformation corresponding to the uncoiling of the collagen molecule, (ii) a linear regime dominated by molecules gliding, and (iii) a second stiffer elastic regime related to the stretching of the backbone of the tropocollagen molecules until the fibril ruptures. Those authors suggest that both the density and the type of cross-links dictate the stiffness of a large deformation regime by increasing the number of interconnected molecules, while the mechanical properties of the cross-links determine the failure strain and strength of the fibril. These findings reveal that cross-links may play an essential role in creating an interconnected fibrillar material of tunable toughness and strength [29].

The membranes selected for this study included native and synthetic collagens. The microstructure of these was unequivocally different, making it impossible to evaluate the influence of the collagen fibers' orientation on the results [15, 26].

Thompson and Czernuszka examined the effect of cross-linking of collagen on the mechanical properties of the polymer using 2 techniques: induction by glutaraldehyde and application of a combination of dehydrothermal treatment and cyanamide. These authors examined the materials, before and after cross-linking. Their results showed that cross-linking itself increased the elastic modulus, reduced the strain to failure, and had little effect on the fracture stress (ultimate tensile strength) of collagen under the experimental conditions [30].

In search for an ideal GTR/GBR membrane, the characteristics of the mechanical and physical properties must be considered in order to avoid membrane collapse and to improve the ease of handling and placement. Recent advances in the development of GTR/GBR membranes with the desirable features and properties have been made by combining natural and synthetic polymers with or without therapeutic drugs or biologic mediators [31]. The rationale of having a regenerative membrane with a graded structure is based on the principle that one can tailor the properties of the different layers to design a membrane that will retain its structural, dimensional, and mechanical integrity long enough to enhance periodontal regeneration. However, most of these methods result in membranes with reduced efficacy for clinical application due to high density and difficulty in handling as well as a heterogeneous, nonuniform degradation rate [32].

Further investigation of the correlation between membrane structure and density, as well as of the biological, clinical, and functional qualities of membranes in current use, is recommended.

## 5. Conclusions

This research presents data on the mechanical qualities of clinically used collagen membranes.

Collagen membrane density has a significant positive influence upon resistance to tearing. Dry membranes exhibited significantly higher mechanical resistance to tensile forces than wet ones. Among the 3 membranes examined, Remaix™ was significantly more resistant to tensile force than Bio-Gide® and Ossix Plus®, while Ossix Plus® was significantly more fragile than Remaix™ and Bio-Gide®.

## Figures and Tables

**Table 1:** Mean values of physical data of the tested membranes.

Mean ± SD	Bio-Gide dry	Bio-Gide wet	Remaix dry	Remaix wet	Ossix Plus dry	Ossix Plus wet
Thickness (mm)	0.4 ± 0.11a	0.44 ± 0.11a	0.29 ± 0.03b	0.28 ± 0.04b	0.26 ± 0.03c	0.27 ± 0.02c
Volume (mm^3^)	30.50 ± 7.98a	33.17 ± 8.5a	22.24 ± 2.22b	21.54 ± 2.71b	18.84 ± 2.22c	19.92 ± 2.08c
Density (mg/mm^3^)	0.33 ± 0.03a	0.3 ± 0.03a	0.43 ± 0.028b	0.4 ± 0.03b	0.22 ± 0.03c	0.23 ± 0.02c

Groups with different letters per material and between materials are significantly different (*p* < 0.05).

**Table 2:** Correlations between the membranes' physical parameters and the measured mechanical results.

Mechanical results/physical parameters	Max. load (N)		Max. extension (mm)		Energy (J)		Max. stress (MPa)	
	Dry	Wet	Dry	Wet	Dry	Wet	Dry	Wet
Surface area (mm^2^)	0.26	0.46^*∗∗*^	0.42^*∗*^	0.51^*∗∗*^	0.33	0.42^*∗*^	0.10	0.35
Average thickness (mm)	0.30	0.16	0.35	0.02	0.25	0.02	−0.11	−0.19
Volume (mm^3^)	0.31	0.19	0.37^*∗*^	0.06	0.27	0.05	−0.10	−0.16
Density (mg/mm^3^)	0.78^*∗∗*^	0.85^*∗∗*^	0.91^*∗∗*^	0.90^*∗∗*^	0.92^*∗∗*^	0.84^*∗∗*^	0.68^*∗∗*^	0.90^*∗∗*^
Cross-sectional area (mm^2^)	0.68^*∗∗*^	0.17	0.36^*∗*^	0.04	0.26	0.03	−0.11	−0.18

*N* = 30, all examined type of membranes, wet or dry. ^*∗*^*p* < 0.05, ^*∗∗*^*p* < 0.01.

## Data Availability

The raw data used to support the findings of this study are included within the supplementary information.

## Abbreviations

BTB: Biological tissue barriers
GTR: Guided tissue regeneration
GBR: Guided bone regeneration
PTFE: Polytetrafluoroethylene
ECM: Extracellular matrix
TF: Tensile force
FGM: Functionally graded membrane
PLCL: Poly (d,l-lactide-co-caprolactone).

## Supplementary Materials

Supplementary Materials The data include the physical measurements (e.g., weight, length, and density) of each sample of the examined membrane and the mechanical measurements of the tensile test. The averages were included too. B, Biogide; O, Ossix-plus; R, Remaix, wet/dry (no tags).
